# Co-Circulation of SARS-CoV-2 and Other Respiratory Pathogens in Upper and Lower Respiratory Tracts during Influenza Season 2022–2023 in Lazio Region

**DOI:** 10.3390/microorganisms11092239

**Published:** 2023-09-05

**Authors:** Licia Bordi, Antonella Vulcano, Giuseppe Sberna, Marino Nonis, Paolo Giacomini, Fabrizio Maggi, Carla Fontana, Eleonora Lalle

**Affiliations:** 1 Laboratory of Virology and Biosafety Laboratories, National Institute of Infectious Diseases “L. Spallanzani” IRCCS, 00149 Rome, Italy; licia.bordi@inmi.it (L.B.); giuseppe.sberna@inmi.it (G.S.); fabrizio.maggi@inmi.it (F.M.); eleonora.lalle@inmi.it (E.L.); 2 Laboratory of Microbiology and Biological Bank, National Institute of Infectious Diseases “L. Spallanzani” IRCCS, 00149 Rome, Italy; antonella.vulcano@inmi.it; 3 Health Management Direction, National Institute of Infectious Diseases “L. Spallanzani” IRCCS, 00149 Rome, Italy; marino.nonis@inmi.it (M.N.); paolo.giacomini@inmi.it (P.G.)

**Keywords:** co-infection, respiratory pathogens, syndromic panel, respiratory tract infections, SARS-CoV-2

## Abstract

Lower respiratory tract infections (LRTIs) occur when there is a lower airway tract infection. They are well-known for increasing the susceptibility of patients to bacterial/fungal co-infections and super-infections. In this study, we present the results of our investigation, which involved 381 consecutive patients admitted to our hospital during the Influenza season from October 2022 to April 2023. Among the 381 specimens, 75 were bronchoalveolar (BAL), and 306 were nasopharyngeal swabs (NPSs). Notably, 34.4% of the examined samples tested positive for SARS-CoV-2. Of these, we observed that 7.96% of NPSs showed positivity only for other respiratory viruses, while a substantial percentage (77%) of BAL specimens exhibited positive results only for bacterial co-infections. The results of our study not only confirm the importance of co-infections in COVID-19 but also emphasize the significance of utilizing rapid diagnostic tests (RDTs) for the timely diagnosis of LRTIs. In fact, RDTs allow for the identification of multiple pathogens, providing clinicians with useful and timely information to establish effective therapy.

## 1. Introduction

Lower respiratory tract infections (LRTIs) pose a significant concern in both community and hospital settings due to their high rate of morbidity and mortality [1]. Difficulties in diagnosing LRTIs, such as distinguishing between viral and bacterial infections, further complicate their management [2]. Additionally, co-infections are common [3]. When bacterial infections are present, the antimicrobial treatment challenge is exacerbated by the growing global problem of antimicrobial resistance. Severe acute respiratory syndrome coronavirus-2 (SARS-CoV-2) has served as a significant example of LRTIs and has resulted in an illness known as COVID-19. The SARS-CoV-2 virus damages the respiratory epithelial lining, and its inflammatory and immune dysregulation properties contribute to the development of co-infections with bacteria or fungi [4]. Specifically, bacterial co-infection has been identified as the primary cause of death during pandemics, rather than direct viral insult [4]. On the other hand, the COVID-19 pandemic has had a profound impact on the circulation, seasonality, and burden of respiratory viruses [5]. Recently, Fan and colleagues published a retrospective study performed in China in the period December 2022–January 2023, during the Omicron BA.5.2/BF7 wave [6]. The study showed that 21.5% of 545 SARS-CoV-2-positive patients were co-infected with another respiratory pathogen, including 20.2% of bacteria and 1.3% of viruses. The authors underlined that, among the bacterial infections, more than half were multiple mixed bacterial infections, and about 30% of patients with bacterial co-infections had a severe COVID-19 diagnosis, suggesting that the simultaneous presence of bacteria could influence the morbidity and severity of COVID-19 [6]. Moreover, a recent meta-analysis has highlighted the significant role of fungal-bacterial co-infections and super-infections in COVID-19 patients [7]. Thus, it is important to note that a chest X-ray suggesting a bacterial co-infection indicates the need for immediate hospitalization, especially for immunocompromised patients. For these individuals, initiating empiric antibiotic therapy should also be considered. Furthermore, the prevalence of co-infections and super-infections among hospitalized COVID-19 patients may have a considerable impact on diagnosis and treatment. Therefore, it becomes essential to verify the presence of fungal and bacterial co-infections in COVID-19 patients [7]. In this regard, the syndromic diagnostic approach can represent a true game-changer. In this work, we present a retrospective analysis of the results obtained from the routine diagnosis of respiratory pathogens in upper and lower respiratory tract samples from subjects undergoing molecular investigation for suspected respiratory infections.

Our observations were conducted between the autumn of 2022 and the spring of 2023, during the Influenza season. The data demonstrate the significant role played by the bacterial component in lower respiratory tract infections. In contrast, it appears to be less relevant in upper respiratory tract infections, where the virological etiology predominates.

## 2. Materials and Methods

### 2.1. Study Population and Sample Collection

This study included 381 samples taken from 381 patients [males: 217 (56.96%), median age: 69 (min 2–max 99); females: 164 (43.04%), median age: 71 (min 4–max 99)] who accessed the National Institute for Infectious Diseases “L. Spallanzani” and were positive for respiratory pathogens during the Influenza season (October 2022–April 2023) (Table 1). Among the 381 specimens, 75 were bronchoalveolar (BAL) and 306 were nasopharyngeal swabs (NPSs). Table 2 shows the patients’ diseases on admission to our facility.

### 2.2. Molecular Assays

Undiluted/untreated BAL samples were analyzed using the BIOFIRE^®^ FILMARRAY^®^ Pneumonia Panel plus (BFAPP) (bioMérieux Italia Spa, Bagno a Ripoli, Firenze, Italia) run on the BIOFIRE^®^ FILMARRAY^®^ System (bioMérieux). Pneumonia Panel plus is a multiplexed nucleic acid test for the simultaneous detection and identification of multiple respiratory viral and bacterial nucleic acids, as well as selected antimicrobial resistance genes. The panel detects 18 bacteria (11 Gram-negative, 4 Gram-positive, and 3 atypical): *Acinetobacter calcoaceticus-baumannii* complex, *Enterobacter cloacae*, *Escherichia coli*, *Haemophilus influenzae*, *Klebsiella aerogenes*, *Klebsiella oxytoca*, *Klebsiella pneumoniae* group, *Moraxella catarrhalis*, *Proteus* spp., *Pseudomonas aeruginosa, Serratia marcescens*, *Staphylococccus aureus*, *Streptococcus agalactiae*, *Streptococcus pneumoniae*, *Streptococcus pyogenes*, *Legionella pneumophila*, *Mycoplasma pneumoniae*, and *Chlamydophila pneumoniae*; 7 antibiotic resistance markers (specifically, CTX-M, KPC, NDM, Oxa48-like, VIM, IMP, mecA/mecC, and MREJ), and 9 viruses that cause pneumonia and other lower respiratory tract infections: Influenza A, Influenza B, Adenovirus, Coronavirus, Parainfluenza virus, Respiratory Syncytial virus, Human Rhinovirus/Enterovirus, Human Metapneumovirus, and Middle East Respiratory Syndrome Coronavirus (MERS-CoV). Results are reported semi-quantitatively, with bins representing approximately 10^4^, 10^5^, 10^6^, or ≥10^7^ genomic copies of bacterial nucleic acid per milliliter (copies/mL) of specimen. All steps, from nucleic acid extraction to the final pathogen detection, were carried out automatically. The kit’s sample swab was utilized to dispense the appropriate amount of SPU and/or BAL into the cartridge, following the manufacturer’s instructions. In brief, approximately 200 μL of the sample was collected using a flocked swab and transferred to a sample injection vial. It was then mixed with the provided sample buffer. The resulting solution was loaded into the FilmArray pouch, which, in turn, was placed on the FilmArray platform. The preparation of each cartridge required approximately 2 min of hands-on time, while the run time was around 1 h and 15 min.

Analyses of NPS samples were performed by the QIAstat-Dx Respiratory SARS-CoV-2 Panel (Qiagen s.r.l, Germantown, MD, USA) on the QIAstat-Dx Analyzer. The panel is a multiplexed nucleic acid real-time PCR test intended for the qualitative detection and differentiation of the nucleic acid of 21 viral and bacterial respiratory targets for common pathogens causing respiratory infections, including SARS-CoV-2. This panel can detect the following organism types and subtypes: Adenovirus, Coronavirus 229E, Coronavirus HKU1, Coronavirus NL63, Coronavirus OC43, SARS-CoV-2, Human Metapneumovirus A + B, Influenza A, Influenza A H1, Influenza A H3, Influenza A H1N1/pdm09, Influenza B, Parainfluenza virus 1, Parainfluenza virus 2, Parainfluenza virus 3, Parainfluenza virus 4, Rhinovirus/Enterovirus, Respiratory syncytial virus A + B, *B. pertussis*, *C. pneumoniae*, and *M. pneumoniae*. Also, for this platform, all steps, from nucleic acid extraction to the final pathogen detection, were carried out automatically. Universal Transport Medium was used to collect NPSs, and 300 μL were used to perform the test.

### 2.3. Statistical Evaluation

To determine the potential statistical significance of the differing co-infection distributions observed within the two groups (SARS-CoV-2 positive and negative) for each sample type (NPS and BAL), we utilized the chi-square test and examined the resulting *p*-values. We interpreted values of 0.05 or below as indicators of statistical significance.

### 2.4. Culture

Bacterial culture remains the gold-standard method for the isolation and detection of respiratory pathogens of the upper and lower respiratory tracts, including atypical bacteria. The BAL samples were treated with Sputasol buffer solution (TermoFisher, Gloucester, UK) to fluidize the mucus samples. Part of the BAL secretion sample was diluted 1:1 (*v*/*v*) in this buffer, vortexed, and shaken for 15 min at room temperature. Ten microliters of the treated sample were inoculated on different types of agar plates: Sheep blood agar, Chocolate agar, MacConkey’s agar, Mannitol salt agar, and Sabouraud Dextrose Agar (37 °C, 5% CO_2_) for 24 h. At the end of the incubation, the plates were examined for the presence of significant pathogens. If they were negative, they were kept for further observation for up to 5 days. A positive culture was reported if the potential bacterial pathogen exhibited growth ≥ 10^3^ CFU/mL. The growth of bacterial colonies was monitored, and the identification of bacterial species was performed using the MALDI-TOF assay (MALDI TOF Syrius; Bruker Daltonics, Bremen, Germany), while antimicrobial susceptibility testing (AST) was performed using the Phoenix system (Becton Dickinson; Sparks, MD, USA). The ASTs were interpreted according to EUCAST clinical breakpoints v 10.0, v 11.0, and vs. 12.0, respectively, for 2020, 2021, and 2022 [8,9,10].

The identification of carbapenemase-producing *Enterobacterales* (CPE) was performed using immunochromatographic assays (NG-Test^®^ CARBA 5 (NG Biotech, Guipry, France)) that recognized the epitopes of carbapenemases via antigen–antibody reactions on chromatographic paper and were able to simultaneously detect five major carbapenemases (KPC, NDM, VIM, IMP, and OXA-48-like carbapenemases).

## 3. Results and Discussion

**Figure 1:**
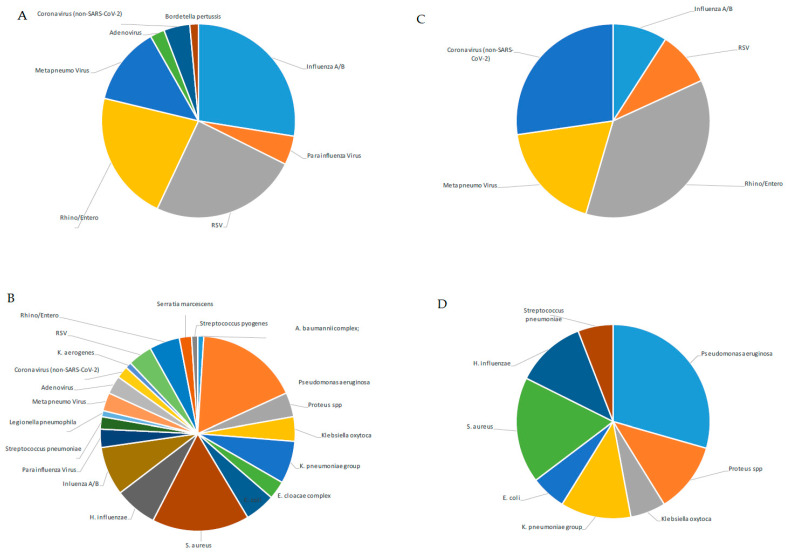
Percentage of detected pathogens in NPS and BAL samples of SARS-CoV-2-negative or -positive patients. Percentage of detected pathogens in 193 NPS (**A**) and in 57 BAL samples (**B**) of SARS-CoV-2-negative patients; percentage of detected pathogens in 113 NPS (**C**) and in 18 BAL samples (**D**) of SARS-CoV-2-positive patients.

From a retrospective analysis of laboratory records, 131 of 381 samples (34.4%) were SARS-CoV-2-positive, and 250 of 381 (65.6%) were negative for SARS-CoV-2 but positive for at least one other respiratory pathogen (Table 3). When the 131 SARS-CoV-2-positive samples were examined, we observed that 9 of 113 NPSs (7.96%) were also positive for other respiratory viruses (Rhino/Enterovirus: 44.4%; Human Coronavirus non-SARS-CoV-2: 33.3%; MPV 22.2%; Flu A/B and RSV 11.1%, Figure 1C), while a high percentage of BAL samples (14/18; 77%) gave positive results only for bacterial co-infections (*P. aeruginosa*: 35.7%, *S. aureus*: 21.4%, *H. influenzae* and *K. pneumoniae*: 14.2%, *Proteus* spp.: 14%; *K. oxytoca*: 7.14%; Figure 1D).

Furthermore, when these samples were cultured to evaluate the viability of the pathogens, it was noted that pathogens with a microbial count of ≤10^4^ copies/mL (cp/mL) were not confirmed by the cultural method. Particularly in cases where two or three pathogens were identified, the culture only confirmed the presence of those with high bacterial loads > 10^5^, as detected by the BFAPP. This trend was particularly evident for *H. influenzae*.

Specifically, among the group of SARS-CoV-2-negative patients, we observed that, out of 16 samples, the culture confirmed the presence of the pathogens detected by BFAPP in 13 samples. However, one sample that tested positive for *H. influenzae* (≤10^4^ cp/mL) was found to be culture-negative. In one instance, we observed the growth of *Enterobacter hormachei*, while BFAPP detected an *E. cloacae* complex. Nevertheless, this discrepancy can be explained by the MALDI TOF’s greater discriminating ability. In two cases (both involving BAL samples with multiple pathogens detected by BFAPP), the growth of *S. aureus* was not evident in the culture, despite the pathogen being detected at loads of 10^5^ and 10^6^, respectively. Lastly, one BAL showed a real incongruence, as the culture was positive for *Candida tropicalis*, while BFAPP detected the presence of two Gram-negative fermenters (*E. coli* and *E. cloacae*).

Considering the culture results in the group of samples collected from SARS-CoV-2-positive patients, five were determined to be negative (probably due to the co-infection with 1–2 pathogens detected by the BPAPP, but exhibiting bacterial loads ≤ 10^5^), while nine fully confirmed the observed positivity. The identified gene markers (*bla*_KPC_, *mec*A, and *bla*_CTX-M_) were also confirmed in the isolates obtained from the cultures. Overall, our results support the hypothesis of Fan and colleagues that the severity of COVID-19 is associated more with bacterial co-infections than with viral ones [6,11,12,13,14]. In fact, in BAL samples associated with more severe conditions, a high proportion of bacteria was detected (77%), while no viral co-infection was observed. This differs from the upper respiratory tract, where a relatively high proportion of Rhino/Enterovirus, Human coronavirus non-SARS-CoV-2, and MPV and a low proportion of Flu A/B and RSV were detected. Moreover, the primary variant of the virus observed during our study was Omicron. However, considering the ongoing emergence of new variants, it would be intriguing to establish a correlation between the distinctive SARS-CoV-2 variants and the co-infections that were observed. This could provide valuable insights in a subsequent study [15,16].

It is noteworthy that in the BAL group of SARS-CoV-2-negative patients, co-infections with two bacterial pathogens were observed in 16 patients. However, no prevalent association between microorganisms was found. On the contrary, in the BAL group of SARS-CoV-2-positive patients, only five cases of two or more bacterial co-infections were observed. Similarly, no prevalent associations between pathogens were found in this case.

**Figure 2:**
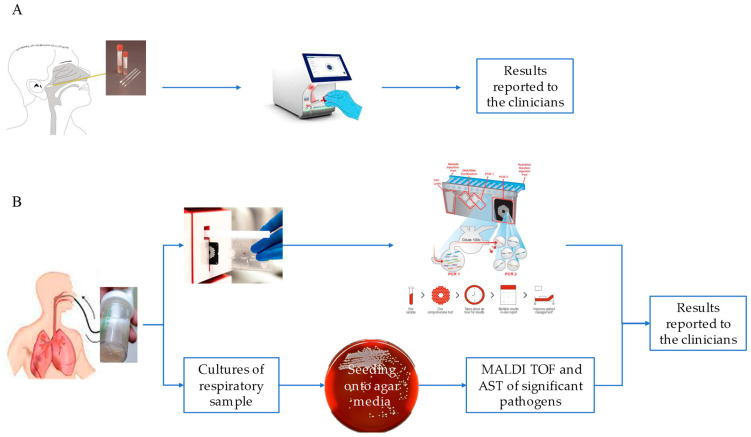
NPS and BAL workflows. NPS workflow using the QIAstat-Dx Respiratory SARS-CoV-2 panel (**A**). Workflow on a BAL sample using the FilmArray Pneumonia Panel plus (**B**).

Moreover, we extended the analysis to the 250 samples that were negative for SARS-CoV-2 but positive for other respiratory pathogens. We observed a different distribution of viruses detected in the upper respiratory tract (193 NPSs) with respect to SARS-CoV-2-positive samples. Those with a higher percentage were Influenza and RSV (29.5% and 26.4%, respectively), and those with a lower percentage were Rhino/Enterovirus, MPV, Human Coronavirus non-SARS-CoV-2, and ADV (23%; 13.9%, 4%, and 2.59%, respectively, Figure 2A). Notably, when looking at SARS-CoV-2-negative samples, bacteria were poorly represented in NPSs (*B. pertussis*: 1.5%, Figure 1A), while a broad range of both bacterial and viral respiratory pathogens was found in the lower respiratory tract (Figure 1B), unlike that observed in the same district of SARS-CoV-2-positive samples. However, a possible limitation in our findings could be that the NPS and BAL samples were analyzed separately, using kits specific for detection of pathogens of the upper (QIAstat-Dx Respiratory SARS-CoV-2 Panel) and lower respiratory tracts (BIOFIRE^®^ FILMARRAY^®^ Pneumonia Panel plus), according to a different workflow (summarized in Figure 2). Therefore, the QIAstat-Dx Respiratory Panel system detects a smaller and different assortment of bacterial pathogens than the FILMARRAY^®^ Pneumonia Panel plus.

Our study, aimed at describing the co-circulation of SARS-CoV-2 with other respiratory pathogens in different respiratory districts in patients with Influenza-like illnesses during the Influenza season (October 2022–April 2023), highlights the presence of a substantial percentage (77%) of bacterial co-infections in the lower respiratory tract of SARS-CoV-2-positive patients, with viral co-infections being completely absent. On the contrary, when analyzing the upper respiratory tract, only viral co-infections were detected (7.96%). Analyzing the 131 samples from SARS-CoV-2-positive patients without distinguishing between the upper and lower respiratory tracts, the percentages of bacterial and viral co-infections drop to 10.6% and 6.8%, respectively, in line with data described in other studies [17,18].

Finally, the observed values of the chi-square test and the *p*-value in the comparison of the frequency distributions of co-infections observed in SARS-CoV-2-positive and -negative patients, respectively, for upper and lower respiratory tract infections, were 20.09 with a *p*-value of 0.000479, and 64.61 with a *p*-value of 0.000013. These values confirm that the different observed co-infection associations are statistically significant. However, the different distribution of the pathogens observed is linked neither to the patients’ clinical conditions nor to their ages at admission.

Overall, our results confirm the utmost importance of recognizing co-infection in patients with COVID-19, since bacterial co-infections represent the most common complications and could be considered critical risk factors influencing morbidity and severity. A prompt differential diagnosis for respiratory viruses and bacteria is crucial to directing therapy and limiting the inappropriate use of antibiotics [19,20,21]. Furthermore, the detection of some relevant markers of resistance is very useful for establishing an empirically targeted therapy for the treatment of patients at risk of respiratory failure [2,7,22]. Respiratory infections can be caused by a variety of pathogens, including bacteria and viruses, usually presenting with roughly similar clinical signs and symptoms. Rapid and accurate determination of the presence or absence of potential causative agents helps make timely decisions about treatment, hospitalization, and infection management, greatly supporting improved antimicrobial stewardship and other important public health initiatives.

## Figures and Tables

**Table 1:** Description of NPS and BAL samples analyzed.

	Total		SARS-CoV-2 Positive Other Respiratory Pathogens Positive		SARS-CoV-2 Negative Other Respiratory Pathogens Positive		SARS-CoV-2 Positive Other Respiratory Pathogens Negative	
	NPS	BAL	NPS	BAL	NPS	BAL	NPS	BAL
Number of patients (%)	306 (80.31)	75 (19.67)	9 (2.36)	14 (3.67)	193 (50.66)	57 (14.96)	104 (27.30)	4 (1.05)
Male (%)	173 (56.54)	44 (58.67)	4 (44.44)	10 (71.43)	103 (53.37)	33 (57.89)	66 (63.46)	1 (25.00)
Female (%)	133 (43.46)	31 (41.33)	5 (55.56)	4 (28.57)	90 (46.63)	24 (42.11)	38 (36.54)	3 (75.00)
Median age (years) (±SD)	71 (±19.7)	66 (±17.8)	71 (±20.7)	76.5 (±15.1)	67 (±21.9)	63 (±18.2)	74.5 (±13.9)	70.5 (±10.6)

SD: Standard deviation.

**Table 2:** Percentage of patients’ diseases on admission to our facility.

	NPS	BAL
Renal failure	0.8	-
Respiratory failure	0.8	-
Cerebral ischemia	0.8	-
Cytomegalic disease	0.8	-
Alveolar and parietoalveolar pneumopathies	0.8	-
Severe sepsis	0.8	-
Pleural effusion	0.8	-
Complications of liver transplantation	1.7	-
Influenza	1.7	-
Meningitis	2.5	-
HIV-1 complications	5.0	-
Bacterial pneumonia	5.0	6.7
Viral pneumonia	5.9	-
Suspected tuberculosis	5.9	6.7
COVID-19 pneumonia	24.2	-
COVID-19 respiratory distress syndrome	42.5	86.6

**Table 3:** Presence of respiratory pathogens (non-SARS-CoV-2) in NPS and BAL samples.

Pathogens	Percentage			
	NPS (SARS-CoV-2 Negative)	NPS (SARS-CoV-2 Positive)	BAL (SARS-CoV-2 Negative)	BAL (SARS-CoV-2 Positive)
Influenza A/B	29.53%	11.11%	10.67%	-
Parainfluenza Virus	5.18%	-	4.00%	-
RSV	26.42%	11.11%	5.33%	-
Rhino/Entero	23.32%	44.44%	6.67%	-
Metapneumo Virus	13.99%	22.22%	4.00%	-
Adenovirus	2.59%	-	4.00%	-
Coronavirus (non-SARS-CoV-2)	4.66%	33.33%	2.67%	-
*B. pertussis*	1.55%	-	-	-
*A. baumannii complex*	-	-	1.33%	-
*P. aeruginosa*	-	-	22.67%	35.71%
*Proteus* spp.	-	-	5.33%	14.28%
*K. oxytoca*	-	-	5.33%	7.14%
*K. pneumoniae group*	-	-	9.33%	14.28%
*E. cloacae complex*	-	-	4.00%	-
*E. coli*	-	-	6.67%	7.14%
*S. aureus*	-	-	21.33%	21.43%
*H. influenzae*	-	-	9.33%	14.28%
*S. pneumoniae*	-	-	2.67%	7.14%
*L. pneumophila*	-	-	1.33%	-
*K. aerogenes*	-	-	1.33%	-
*S. marcescens*	-	-	2.67%	-
*S. pyogenes*	-	-	1.33%	-

## Data Availability

The data used and/or analyzed during the study are available from the corresponding author on reasonable request.
